# Digital Companion Choice to Support Teachers’ Stress Self-management: Systematic Approach Through Taxonomy Creation

**DOI:** 10.2196/32312

**Published:** 2022-02-16

**Authors:** Julia B Manning, Ann Blandford, Julian Edbrooke-Childs

**Affiliations:** 1 Interaction Centre Department of Computer Science University College London London United Kingdom; 2 Institute of Healthcare Engineering University College London London United Kingdom; 3 Evidence-based Practice Unit University College London and Anna Freud Centre London United Kingdom

**Keywords:** digital technology, digital health, psychological treatment, stress, self-management, mobile phone

## Abstract

**Background:**

There are thousands of digital companions designed for emotional well-being and stress, including websites, wearables, and smartphone apps. Although public evaluation frameworks and ratings exist, they do not facilitate digital companion choice based on contextual or individual information, such as occupation or personal management strategies.

**Objective:**

The aim of this study is to establish a process for creating a taxonomy to support systematic choice of digital companions for teachers’ stress self-management.

**Methods:**

We used a 4-step study design. In step 1, we identified the dimension of stress self-management and strategic classifications. In step 2, we identified the dimension of the digital techniques and conceptual descriptions. In step 3, we created 6 criteria for the inclusion of digital companions. In step 4, we used the taxonomy framework created by steps 1 and 2 and populated it with digital companions for stress self-management, as identified in step 3.

**Results:**

First, in the dimension of stress self-management, we identified four classes of strategies: educational, physiological, cognitive, and social. Second, in the digital techniques dimension, we derived four conceptual descriptions for the digital companions’ mechanisms of action: fostering reflection, suggesting treatment, peer-to-peer support, and entertainment. Third, we created six criteria for digital companion inclusion in the taxonomy: suitability, availability, evaluation, security, validity, and cost. Using the taxonomy framework and criteria, we populated it with digital companions for stress management ahead of presentation to teachers in a stress study workshop.

**Conclusions:**

The elements of our approach can be generalized as principles for the creation of taxonomies for other occupations or conditions. Taxonomies such as this could be a valuable resource for individuals to understand which digital companion could be of help in their personal context.

## Introduction

### Background

Self-care digital health smartphone apps, websites, and wearables, referred to collectively in this paper as digital companions, are ubiquitous, but understanding which of these will best support individual needs in a given context is complex. The selection presented to the potential user is immense, with at least 10,000 digital companions targeting behavioral and mental health [1], and the existing approach to digital companion selection is often opportunistic. The availability of mental health apps is hampered by high turnover: 50% of search results change within 4 months, with an app being removed every 2.9 days from web-based platforms [2] and more than 200 health apps being added every day to app stores [3]. Routes to adoption of digital companions for psychological support include recommendations from health professionals [4], although a US study found social media, personal searches, and word of mouth to be more common access routes [5].

User recommendation on app stores is another common route, but it has its limitations, such as including different types and amounts of coverage. In addition, the sources of these reviews are unknown. Taking the reviews at face value, a more detailed exploration of user recommendations of psychological apps has been achieved by machine learning sentiment analysis, revealing the top positive and negative themes for user satisfaction [6]. High cost, app instability, low quality content, and privacy or security concerns were the most common dissatisfaction themes. Tracking, outcome visualization and analytics, and content quality and variety were the most common satisfaction themes. Another study on anxiety apps alone also revealed that price negatively affects adoption, whereas ratings and reviews positively affect downloads, but only up to a point [7]. We also know that app descriptions influence adoption but can be unhelpful. Potentially stigmatizing labeling such as app titles that imply a diagnosis for a mental health condition can constrain access or even cause harm [8]. Some apps use scientific language in their descriptions to verify their clinical validity. However, a study of 73 popular mental and emotional health apps found that although 44% used such language, only 2 apps provided direct scientific evidence associated with app use [9].

More recent studies have begun to elucidate some relevant information on the types of use for technology. One small survey recently showed that although smartphone apps were the most used digital companion to support mental health and well-being, they were often used in conjunction with other tools (eg, social media [10]). Importantly, this study showed a relationship between digital companion medium and purpose: apps are used more for guided activities, relaxation, and tracking; social media is used for sharing experiences and gaining personal understanding; and web-based provision is used for daily stress and anxiety management. This survey did not ask about the use of wearables for stress, but the wearable medical device market continues to grow, with 60% growth predicted between 2019 and 2024 to US $27 billion [11]. Early evidence shows that wearables can accurately capture exposure to psychosocial stress in everyday life [12]. Currently, decisions on wearable choice seem to be guided by perceived value, design, and brand [13] rather than by condition management.

Self-management or treatment techniques are often search terms for digital companions, but critically relevant information such as the suitability of the intervention for an individual’s context, occupation, or existing self-management practices are often missing [14]. In meta-analyses of occupational studies where a digital companion had been used to support general well-being [15] or for anxiety, stress, and depression [16], positive effects in these contexts over the short to medium term were noted. However, there is both considerable variation in occupation and little evidence in these studies of any attempt to align an intervention with a particular role or existing individual management strategy. The tendency is simply to trial a digital companion that supports one or more strategies with an occupational cohort, irrespective of the cohort’s existing stress management strategies and preferences.

We know that the contexts in which people live and work influence their use of and ability to use health technology [17-20] and previous research has called for tailoring of health care technologies to specific users [21,22]. Contextual or strategic data and insight could logically aid both choice and strategy and, therefore, the potential efficacy of digital companions and user outcomes. As has been noted in the study by de Korte et al [23], research on digital companions designed to have work-related relevance for the mental and physical health of employees is scarce. In this paper, we present the processes of developing both dimensions for a taxonomy and the population criteria that facilitate the selection of contextually appropriate digital support for stress. We chose to work with teachers and focus on their stress self-management because of the very high prevalence of work-related stress, averaging 2100 cases per 100,000 educators in the United Kingdom in 2018 [24]. There are indications that COVID-19 may have exacerbated primary stressors for teachers [25], but we already know that contextual factors such as school organization and culture are critical factors for teachers’ experience and management of stress [26-30].

Within the context of schools, individual stress management support can be facilitated by digital companions, particularly if teachers have a taxonomy to inform their choice. This paper, therefore, makes the following contributions:

1. The selection of dimensions within which to classify stress self-management and digital health techniques that could offer support
2. The process applied to develop the taxonomy—one that can potentially be adapted and applied in other contexts where digital support is sought for an individual’s health condition to match their practices and values
3. The methodology for populating the taxonomy
4. A populated intervention taxonomy developed for teachers managing stress, with illustrative examples of apps that address teachers’ needs, available at the time of writing

### Related Work

#### Overview

We describe here prior work and evidence that fed into our choice of dimensions, classification, and selection. This includes teachers’ stress self-management research and previous frameworks and taxonomies on the design and selection of technologies.

#### Teacher Self-management of Stress

Approaches to aid teachers in stress management have been drawn from the literature on occupational stress and often applied population wide, although not without acknowledgment that “some (strategies) were unnecessary or differentially effective in individual cases” [31]. There is evidence of benefits to teachers from stress awareness education [32] and physiological interventions including adapted mindfulness and relaxation training [33,34] and exercise [35]. Psychological intervention evidence includes, for example, cognitive behavioral therapy (CBT)–based programs [36,37] and mindfulness embedded in psychoeducation with social support adapted for teachers [38]. Reflective supervision and consultation [39] and environmental adjustment or social support [32] have also been shown to be helpful.

Recent systematic reviews have examined teacher stress interventions and found a greater effect size associated with a longer duration of intervention, but most interventions were guided and not self-managed [40,41]. Those interventions that were self-managed demonstrated positive effects, although these varied in size. Such interventions targeted stress or burnout symptom reduction, including positive psychology through gratitude journaling [42] and CBT-based education through bibliotherapy [37].

#### Digital Companions for Teachers’ Stress Management

Delivering stress management interventions digitally can enable uptake. For example, digital delivery could reduce the cost of provision, improving accessibility and reducing risks of stigma [43], which could be highly relevant to teachers. One tailored eHealth (ie, internet or mobile-delivered health care) randomized controlled trial for teachers used an internet-based problem-solving therapy (a form of CBT). Teachers receiving the CBT intervention reported significantly reduced symptoms of depression as well as a reduction in their perceived stress after the trial (7 weeks) and at 3- and 6-month follow-up [44]. Another study examined stress as a contributor to insomnia among teachers, finding that unguided web-based CBT with psychoeducation among mostly female teachers significantly improved sleep [45]. A recent review of the effectiveness of occupational e-mental health interventions identified only one other study that included education sector personnel [46]. This was a self-administered web-based CBT-based intervention, but the participants also received weekly personalized feedback on the modules. The effect on the reduction in perceived stress across all sectors was large [47].

#### Taxonomy Creation and Digital Technology Selection

##### Overview

We identified 2 approaches in the literature relevant to our goal of creating and populating a taxonomy. One is the evolution of designer- and researcher-focused frameworks, seeking to improve efficacy and evidence. The other is more focused on clinician and consumer adoption.

##### Designer and Researcher Frameworks

Frameworks focused on developing and evaluating technologies have led to better formalizing, detailing, and defining of digital companion design. The persuasive design principles discussed by Fogg [48], expanded further by Oinas-Kukkonen and Harjumaa [49] and complemented by a design model by Ritterband et al [50], all informed the development of the behavior intervention technologies model for developers by Mohr et al [51]. This model, along with other theory-based [14,52] and empirically based [20,53] taxonomies and frameworks, has sought to enable both better conceptual design and easier evaluation of digital companions. The Mobile App Rating Scale (MARS) for designers by Stoyanov et al [53], which has been used extensively in the scientific community, was adapted as a consumer assessment version, uMARS [54]. For this study’s taxonomy, these models informed our consideration of the digital techniques dimension of the taxonomy.

##### Clinician and Individual Frameworks

Both the MARS and the uMARS have been used for evaluating apps, with the latter using less technical language for patients to provide feedback on the engagement, functionality, aesthetics, information, and subjective appreciation of quality and impact. The uMARS allows classic human-computer interaction features and elements to be evaluated to assist design iteration, but it was not created to inform final user adoption. Three other relevant *expert review evaluation frameworks* (Reviews) have been created for users.

The ORCHA (Organisation for Review of Care and Health Apps) model, now paywalled, was specifically designed to inform adoption of mostly apps and has some web-based interventions too. Search is based on the condition or digital companion name. Data privacy, user experience, and clinical assurance are each given a score [55,56].

The two other Reviews focus on psychological health: Mindtools and Psyberguide websites [57]. Psyberguide is a public-facing website that enables a search based on conditions or treatment approaches. The approach taken is that the user understands what concepts or treatment they want to choose (eg, tracking or social support), and the focus is on apps. Both websites publish assessment scores on credibility, user experience, and transparency, although Mindtools does not seem to have been updated since 2017. Psyberguide drew on the MARS framework, incorporating additional privacy and security considerations. The American Psychiatric Association app framework [58,59] has also implemented this. Their framework provides a template for user assessment rather than presenting their own assessment scores. It offers both a quick 8-question *screener* and a much more detailed 5-step, 105-question app evaluation process that allows the end user to judge what is important and a good match. The starting point for this framework is clinical diagnosis, which informs the potential app selection. In theory, their questions could be applied to websites and wearables as well, although this does not appear to have been tested yet.

**Figure 1:**
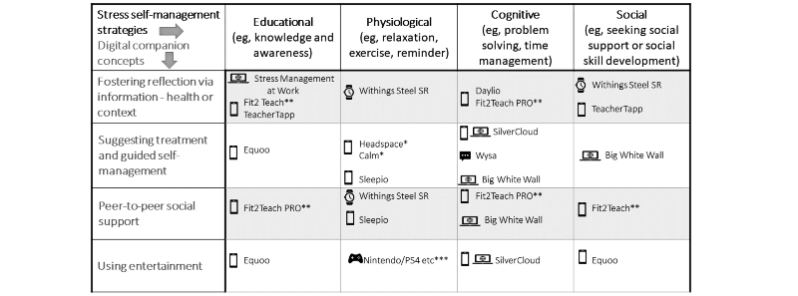
Populated taxonomy with digital stress companion choices for teachers. *Only partial encryption of data **Withdrawn due to lack of updates ***User to provide own device.

The main difference between these scales, Reviews, and frameworks, and our intended approach is the starting point. Our goal was to enable digital companion selection to be framed by someone’s occupation, condition, and self-management behavior. For this, we required a taxonomy derived for teachers and stress from which they could identify their self-management strategy and supportive technology concept and then identify a digital companion that aligned with these to trial in a future study. To achieve this goal, we first required selecting a logical dimension within which to classify stress self-management. Second, we selected a dimension within which to classify digital techniques that could support these strategies. Finally, we created a rationale for digital companion inclusion and the selection of credible candidates. This outcome is illustrated in Figure 1. This paper describes why we chose the dimensions of self-management strategies and digital companion concepts, how we categorized them, and our approach to identify the potential candidates.

## Methodology

**Figure 2:**
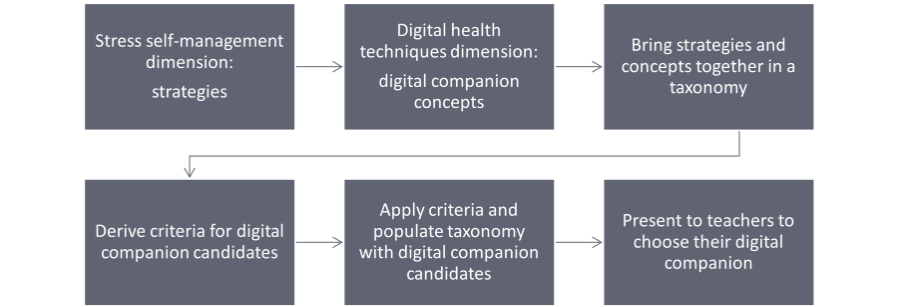
Study design process summary.

The study design process is summarized in Figure 2.

### Study Design

#### Stress Management Dimension

To choose categories for the stress self-management dimension, we initially extracted descriptions from the qualitative data on the experiences of 14 senior teachers interviewed in a previous study [60]. The participants had provided more than 80 accounts of how they managed their stress. These descriptions were complemented by evidence from systematic reviews of occupational stress [23,40,61].

The interventions informed the *PICO* literature search criteria: patient and problem (eg, teacher and stress), intervention (eg, information, tracking, exercise, or mindfulness), comparison (often none), and outcome (eg, identifying, support, management, and reduction). We adapted the narrative method used in other studies [62,63], including checking references of relevant papers, alerts, and citation tracking along with searches of academic databases including PsycINFO, Google Scholar, Cochrane, and PubMed. Literature relevant to teachers’ self-management of stress was reviewed until repetition of themes revealed no further insight. Quality of papers was determined through their being published in peer-reviewed journals.

#### Digital Health Techniques Dimension

For the health techniques dimension, we reviewed the literature on persuasive design, digital health taxonomies, and trends in digital health self-care, again using the snowballing method as described above. We were aware of drawing on the different but complementary cultures of human-computer interaction and health and that their definitions of lifecycles, evaluation and implementation differ [64]. Our interest was in producing conceptual descriptions of mechanisms of action that could support the methods of stress management already identified in the literature and those given by teachers in interviews. These concepts would necessarily comprise elements of design, behavior, and theory, and draw on evidenced deployment of a digital companion for health self-management. Our aim was to create a conceptual description of the prevalent overarching technique or action of the digital companion that could be understood without ambiguity or complexity by the end user.

This approach was chosen for several reasons, including the following: (1) many digital companions use multiple techniques, and we wanted to facilitate choice by the primary featured enabled action and (2) other systematic reviews have overlooked or found a paucity in the description of behavior change techniques, which would make categorization of digital companions by such theory harder to achieve [65,66].

### Technology Selection

To identify candidate digital companions, we took the following steps to inform our decisions:

1. Suitability: we began with digital interventions used by teachers, as described in a previous qualitative study, followed by a review of the literature for other candidates.
2. Availability: we examined whether the technology is accessible on the 2 main mobile operating systems and had been updated within the last 6 months.
3. Evaluation: we checked whether the technology was ranked positively on 1 of 3 expert review evaluation framework (Review) websites for apps and web-based tools (ORCHA [55] and Mindtools [67]) or apps only (Psyberguide [68]) for credibility and evidence base and for user experience.
4. Security: we reviewed the privacy and security policy to assess whether the technology used encryption for data connection and storage (where relevant).
5. Validity: we searched for significant, published positive clinical trial results.
6. Cost: given that the commercial model for apps that are free means very limited access or a trade in personal data, which we did not want to promote, we set a bar of £50 (US $64) annual fee for smartphone and website apps and £150 (US $202) for a wearable.

### Taxonomy Creation

#### Overview

The process of reviewing the existing literature for the creation of stress management and digital techniques dimensions revealed different approaches to classification. Below, we present our findings and rationale for our choice of classification of strategies and concepts and then share the procedure we followed to enable technology selection.

#### Stress Self-management Dimension

##### Overview

We found 3 main approaches to categorize interventions specifically for the support or management of stress experienced by teachers. It is worth emphasizing that the value and goal of this conceptual categorization for our taxonomy was to identify a practical, actionable strategy for the individual [69]. The classification approaches found were as follows: (1) the level targeted by the intervention, (2) the target of intervention, or (3) the intervention strategy. We describe each of these and why we considered the intervention strategy to have the most relevance and explanatory power for the stress dimension.

##### Level of Intervention

Organizational-, individual-organizational–, or individual-level interventions have been frequently described [61,70-73], with an additional level of a classroom-focused approach being noted more recently [74]. The level of the intervention appears to be a way of describing the agent or group responsible for the stress management strategy. For example, the school leadership team or Multi-Academy Trust directors would be at the organizational level. As our focus was on self-management, this categorization would not provide a practical framework for teachers’ own stress management.

##### Target of Intervention

The primary targets of interventions were the stressors themselves, which could be aspects of the work environment, such as maintaining discipline, time pressures, and workload [75]. The corresponding stress reduction strategies would then seek to reduce the occurrence of occupational stress among employees, such as workload reduction. This primary preventive approach for individuals should be the priority and a normal part of organizational management, as has long been argued in the health care sector [76,77]. Although many targets are well described in the teaching literature, they are beyond the control of the individual.

The secondary targets were the perception or responses of the individual person to the stressor itself, and the interventions were preventive or reactive. By targeting the way someone manages or copes with stress, the aim was to modify the individual’s response in a positive way rather than removing the stressor itself. This might include peer support groups or cognitive behavioral techniques.

The tertiary targets of intervention were stress symptoms themselves, such as anxiety, insomnia, or racing heart rate, and the intervention was reactive. The aim of targeting symptoms was to manage or treat the emotional, cognitive, behavioral, or physical changes brought about by stress. Although identifying secondary and tertiary targets enables a better understanding of stress, they do not indicate a set of potential self-management choices. For instance, if a teacher becomes aware that their response to stress is a behavioral habit (both a response and a symptom), such as to start pacing the floor, this knowledge in itself does not provide any signposting to what action an individual can then take to combat the stress. In addition, stress symptoms, such as nervous tics or fatigue, are not always obvious to the individual. Levels and targets of interventions were used in a prior categorization of occupational stress management from general employee work [78,79]; however, for our study’s purposes, this conceptual framework does not always facilitate individual identification of actions that could be taken to self-manage stress.

##### Intervention Strategies

The third approach we identified was stress management strategies or training approaches [41,80-82]. We identified five overarching, nonmutually exclusive categories that could be supported digitally: (1) educational, (2) physiological, (3) situational, (4) cognitive, and (5) social.

The previous strategies have been described as follows: (1) stress awareness and education, (2) relaxation techniques, (3) cognitive coping, (4) biofeedback, (5) meditation, (6) exercise, (7) lifestyle advice, and (8) interpersonal skills training [81]. We considered that several of these could be grouped together along with more detailed activities simply listed as exemplars. Thus, education, awareness, and lifestyle advice were grouped under education; biofeedback, relaxation, meditation, breathing, aerobic activity, or mindfulness were grouped under physiological; and cognitive coping strategies, such as controlling emotions, problem-solving, or time management, were grouped under cognitive.

Social support was mentioned by the authors but was not listed by them as a category. It goes beyond interpersonal skills training embracing socializing and the therapeutic value of peer support [83] and self-enhancing humor [84]. This social element, along with descriptions of social support, has been described in teachers’ stress management research [32,38,41]; hence, we added it as a category. We also noted in the literature some variation in the meaning of mindfulness among educators. This could mean the application of the established 8-week *mindfulness-based stress reduction* program [85,86] or the incorporation as part of a stress reduction program [38] or simply a meditative component of a multi-strategic stress reduction study [29]. Although other authors have used mindfulness-based interventions for categorization [40], the ambiguity in the use of the term meant we decided against using it as a category for strategy.

#### Digital Techniques Dimension

##### Overview

Our aim was to create a concise choice architecture that would be meaningful for potential users. This meaning was established through the description of how a digital companion would provide support.

Other condition-specific intervention reviews demonstrate varying approaches to the classification of technologies. Suijkerbuijk et al [87] categorized dementia interventions by purpose, such as support in daily life, safety, meaningful activities, or communication. Singh et al [88] categorized HIV apps and websites by functionality, such as prevention, testing, and management. These approaches sometimes blended the strategy with the mechanism or contained the mechanism within each function and helped us recognize that the primary focus for our categorization should be the broad mechanism of how the technology technique enabled self-care.

Despite an increasing number of studies on the use of digital companions in the workplace for occupational stress [89], reviews often focus on the type of intervention, such as CBT or mindfulness [16], and grouping them as such [40]. Reviews of the mechanism of action or concepts used by these apps are scarce, and others have noted this lack of detail in persuasive technology design [90]. In addition, reviews of wearables mostly seem to have focused on those for physical activity [91], but others have reported on the incorporation of behavior change technique *clusters* [92]*.* These enabled us to compare and make high-level reconciliation with the motivational affordances described by Orji and Moffatt [93], whose categorization was not always exclusive to one of the condensed descriptions below.

We found that the self-care *opportunities* by Nunes et al [94] essentially conceptualized action-enabled design features which were similar to descriptions given by Klasnja and Pratt [95] for intervention strategies and features. Therefore, we reviewed the descriptions against each other to compare the technique concepts. We then cross-checked them with the descriptions given by Orji and Moffatt [93] to arrive at 5 comprehensive conceptual themes that we now describe as our digital companion concepts.

##### Fostering Reflection by Making Health and Contextual Information Available

Both Klasnja and Pratt [95] and Nunes et al [94] described the ability to track health data first, and we retained the definition by Nunes et al [94] of “fostering reflection by making health and contextual information available.” This data-enabled reflection has been found to be significant for those with severe mental illness [96], bipolar disorder [97], and stress [98], among other psychological conditions.

##### Suggesting Care Activities or Treatment Adjustments and Guided Self-management

The second description of “suggesting care activities or treatment adjustments” by Nunes et al [94] went beyond the mere “increasing accessibility” of health information described by Klasnja and Pratt [95] to actual adjustments that an individual can make. However, this category also needed to explicitly include delivering guided self-management described in the literature on stress, such as directed breathing or a CBT program. Hence, the second category was adapted to *suggesting care activities or treatment adjustments and guided self-management*.

##### Peer-to-Peer Social Support

Nunes et al [94] specifically described a trend as “sharing self-care activities and learning from others with the same chronic condition.” The limitation of this for our purposes was the medical emphasis, but we did want to include the significance of peer relationships. Klasnja and Pratt [95] talked about “leveraging social influence,” capturing the social-sharing concept, building on the social support principles proposed by Oinas-Kukkonen and Harjumaa [49], so we redefined this category as *peer-to-peer social support*.

##### Using Entertainment

Klasnja and Pratt [95] also described using entertainment. This went beyond the gamification techniques recognized by Nunes et al [94], which can be used in the technology design of any of their categories. Participating in a purely fun tech-enabled activity not intentionally designed for symptom management has been shown to reduce stress symptoms [99,100].

##### Involving the Health Care Team

Nunes et al [94] strongly emphasized the patient (not medical) perspective, but 2 of their 5 categories still recognized the shared-care dynamic between patients and their formal and informal carers. Klasnja and Pratt [95] recognized this shared approach but described it under a single form of intervention (involving a health care team), and for our purposes, this sufficed.

**Figure 3:**
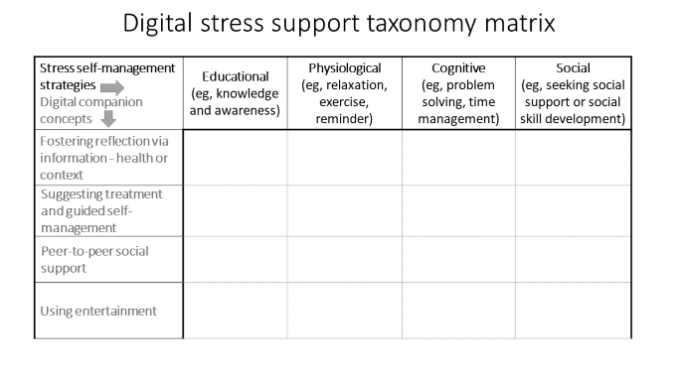
Taxonomy matrix.

For our taxonomy, we did not require the concept of involving the health care team as we were focusing on self-management. Therefore, we brought the 4 digital companion concepts with the 4 stress self-management strategies together in a matrix to give us a taxonomy that could then be the framework for digital companion selection. As a stand-alone taxonomy, this framework provides a structure for anyone seeking to choose a tool to support stress management. Figure 3 depicts this taxonomy.

### Taxonomy Population

To populate the taxonomy, we applied the technology selection steps. This selection process was important for ensuring trustworthy digital companion candidates from which teachers in a subsequent study could make informed choices. The process is summarized in Table 1.

Our starting point was suitability and availability, based on a previous qualitative study exploring teachers’ familiarity and use of digital tools for stress management [60]. This reflected insight into the influence of context to design as described in both usability study methodologies [101] and the person-based approach [20]. Where that did not provide a candidate, we reviewed the literature, the National Health Service (NHS) App Library, Carlo’s behavioral health app review [102], and the scientific literature. Of the 12 apps originally named by teachers, 8 (67%) were available on both iOS and Android platforms (TeacherTapp, Fit2Teach, Headspace, Mindshift, Pacifica [now called Sanvello], Calm, Insight Timer, and Happy not Perfect), but one of these (Fit2Teach) had not been updated for over 2 years. Given that it was uniquely tailored in its approach and that the associated Facebook group had recently been updated, we contacted the developer, but unfortunately, we received no response. Neither Fit2Teach or TeacherTapp had been designed for stress, but both offer education tips and insight, and the opportunity for reflection.

The 2 apps that used diarizing as their prevalent tracking strategy (My Wonderful Days and Now Then Free) were not available on either platform and the other 2 app descriptions were not complete enough for certain identification. The web-based CBT program that had been described by 1 teacher was only available in 1 English county. The wearables used by teachers were Fitbit (Fitbit, Inc) models (Charge, Alta, and Blaze), Samsung Gear 2 (Samsung Electronics Co, Ltd), Polar M340 (Polar Electro Oy), and Apple Watch (Apple, Inc). No other candidate technologies were identified in the literature on teachers’ stress.

We searched for available digital companions within the positive expert review evaluation frameworks (Reviews) but owing to disparities observed between Review assessments [102] and our concern with privacy and safety, we read through all the security and privacy policies. This was also important for all wearables, as none of them were covered in the Reviews. Occasionally, security through encryption was still not evident from the published policy, and in these cases, the developer was emailed for further information.

**Table 1:** Summary of digital companion population process.

Technology selection steps	Rationale
Suitability: qualitative data from occupation and behavior	We began with digital interventions used by teachers as described in data in a previous qualitative study
Availability: verify whether the technology is accessible on the 2 main mobile operating systems and had been updated within the last 6 months	Ensures the technology is available to a wider audience and supported by the developers
Evaluation: search one or more of the expert review evaluation frameworks (Reviews) to see if the technology is ranked positively	Gives professional or third-party view on the credibility, evidence base, and user experience
Security: review the privacy and security policy	Shows whether the data are stored and transmitted securely with encryption to give an indication of risk
Validity: search for research papers on the technology	Enables any trials with the technology to be considered
Cost: assess cost	Considers whether the technology is in budget

Many digital companions have not been tested through trials, so this step (validity) was not a reason to exclude them, especially wearables where data are sparse. Conversely, some popular apps that did not satisfy the safety inclusion criterion had significant published evidence of their efficacy. For these, we presented this scientific evidence as a reason for inclusion, despite no or partial encryption. Finally, cost was considered.

Our final selection of digital companions for presentation to teachers comprised 4 apps named by teachers in the previous study (Headspace, Calm, TeacherTapp, and Fit2Teach), 4 alternative apps sourced from one or more of the Reviews (Equoo, Sleepio, and Daylio), and 1 app from the scientific literature (Wysa, an artificial intelligence–based chatbot). For websites, 1 was sourced from a Review (Big White Wall, now Togetherall Ltd), 1 from the NHS (Stress Management at Work), and 1 from scientific literature (SliverCloud Health). For wearables, 1 was identified from the scientific literature using medical grade data assurance (Withings Steel HR watch, Withings, Inc).

The stress self-management strategies, digital companion concepts, and selected apps were brought together in the taxonomy matrix shown in the introduction in Figure 1 with caveats shown by asterisks.

## Discussion

### Principal Findings

This paper describes the process of creating a context-based framework to facilitate the choice of digital companion intervention. Using the dimension of stress self-management, we created classifications of strategies that were derived from empirical research and the literature. Using the dimension of digital techniques, we created conceptual descriptions of the mechanisms of action of digital companions informed by the literature. Bringing these together in a taxonomy gave the framework that we could populate with digital companions for teachers’ stress self-management according to availability, evaluation, security, validity, and cost. It is a starting structure for the presentation and selection of contextually appropriate digital companions.

Populating the taxonomy presented some significant challenges. The transience of apps or their ratings (availability and evaluation) meant that by the time we came to present our taxonomy to teachers, 1 peer-to-peer–supported CBT website had been removed. Likewise, a highly rated diarizing app had one of its review ratings plummet during our study, although we found no cause for concern on rechecking the privacy policy. Another CBT course with extensive validation through research publications was included, as it had been commissioned by the local NHS in the areas where the teachers we planned to work with were employed. However, when 1 participant tried to access it, a referral from the general practitioner was required, which precluded pure self-management. Some apps we considered were described as designed for stress but included reference to medical conditions such as psychosis and schizophrenia. We were concerned that their inclusion would imply a medical need or that such a diagnostic association could be too sensitive for a study that was focused on occupational stress.

It became clear as we reviewed candidate smartphone apps that many did not offer comprehensive (if any) encryption of data, even those where the funding model required user payment (thus requiring input of more sensitive data). Our search was not exhaustive: that would have been impossible. To ensure candidates in each category, when we were able to reference scientific studies on app efficacy (eg, headspace and calm), it was decided to include them in the taxonomy with the caveat that although widely used, there was no or only partial encryption of stored and/or transmitted user data.

The sequence of application of our selection criteria was affected for wearables because of their cost. Of the 6 different wearables described in the teachers’ study, because of the price, we excluded Samsung Gear 2, Polar 340, and Apple Watch. Obsolescence excluded 2 of the Fitbits (Blaze and Alta), leaving the Fitbit Charge. This failed the encryption requirement being nonspecific and considered external evaluation to be inadequate [103]. Database search, paper retrieval, and website scrutiny enabled us to identify 1 wearable from Withings that satisfied all the set criteria, offered support for 2 of the 4 stress self-management strategies, and fell into the set price bracket.

Importantly, using qualitative field data as a starting point was crucial for identifying digital companions that would not have appeared in a search based on the condition of stress. For example, TeacherTapp was designed as a research tool to voice teachers’ opinions. However, its educational content and sense of peer connection were considered valuable for relieving feelings of stress. Likewise, Fit2Teach, although designed for well-being and work-life balance, was listed under *education* and not under *stress* in app stores.

In a world in which automated or unsubstantiated rating systems are prevalent, there is still a need for autonomous, informed, human decision-making that draws on personal knowledge and understanding [104]. Individuals need to be able to confidently identify their personal preferences to improve their chances of adherence [5]. Improving app selection by context-based condition management and conceptual categorization could logically aid both the adoption and potential efficacy of digital health tools and reduce attrition before the desired outcome. However, our findings illustrate that there is no quick route to informed adoption.

The populated taxonomy was presented to 8 high school midmanagement teachers in workshops to enable them to identify how they currently managed their stress and how it could be supported by digital means. Their chosen digital companions were then used during a planned longitudinal study in the school summer term (during partial COVID-19 lockdown) and on into a serendipitous study in the autumn (where teachers were back in hygiene-adjusted school settings). Of the 8 teachers, 6 (75%) still used their digital companion choice 6 months after beginning. The analysis of these findings will be the subject of a subsequent study*.*

### Limitations

Our review of the literature was not exhaustive, and other research may reveal stress management strategies beyond those we identified. In addition, there could be disagreement on the way that we have grouped or limited the explanatory power of digital companion concepts or that they are relevant for conditions other than stress. Further research will be able to substantiate whether these issues are significant.

We have already noted in our process and discussion that the selection of technology can never be complete and is only ever a reflection of what apps and information are available at the time of the search. In addition, our starting point for apps was a previous small study where the participants had self-selected; a different or wider cohort could have produced other findings. There is no circumventing the reality that populating a taxonomy will always have to be revisited at the time of use.

Another limitation of our approach is potentially in embedding the notion that dealing with or coping with workplace stress is just the responsibility of the individual. This individualized approach can place a profound burden on a teacher as it fails to acknowledge the complexity of the origins of stress [105]. It is not our intention to imply that managing stress is only the responsibility of the individual, and through our context-based approach, we acknowledge the structural and environmental influences, in addition to the sociocultural factors within a school.

### Conclusions

There is no quick and easy solution to identifying a safe, efficacious, contextually, and individually appropriate app, website, or wearable to support self-management of health, well-being, or a specific health condition. Evaluation frameworks are valuable and evolving but would benefit from complementary information for users to be able to identify their preferences and consider whether the technology on offer fits their current behaviors or contexts.

If an individual can use a taxonomy to identify their preferred management strategy and, from there, make an informed selection of a digital companion for support, the user starts from a strong position. We hope that these procedures can generally inform professionals seeking to facilitate the selection of a digital companion for an individual’s self-management of a named health or well-being condition. We also hope that our populated taxonomy can be a specific starting point for teachers’ digital companion–supported stress self-management, and one that can be refreshed through repopulation in the future.

## Abbreviations

CBT: cognitive behavioral therapy
MARS: Mobile App Rating Scale
NHS: National Health Service
ORCHA: Organisation for Review of Care and Health Apps
